# Supplementary material for: Impact of host factors and invasive meningococci on bacterial adhesion, proliferation, primary nasal epithelial barrier function, and immune response
Source: Microbiol Spectr. 2025 Jun 30;13(8):e00141-25. doi: 10.1128/spectrum.00141-25 (PMC12323337; doi:10.1128/spectrum.00141-25)

**Supplemental figure 1:** (A) TEER fold change for six additional strains (yellow) on donors D5, D6 and D7. In red and blue are the data as in fig 1. (B) TEER fold changes for additional strains (green) do not differ from previously tested strains (blue). Mean donor (A) or strain (B) fold change with median is shown.


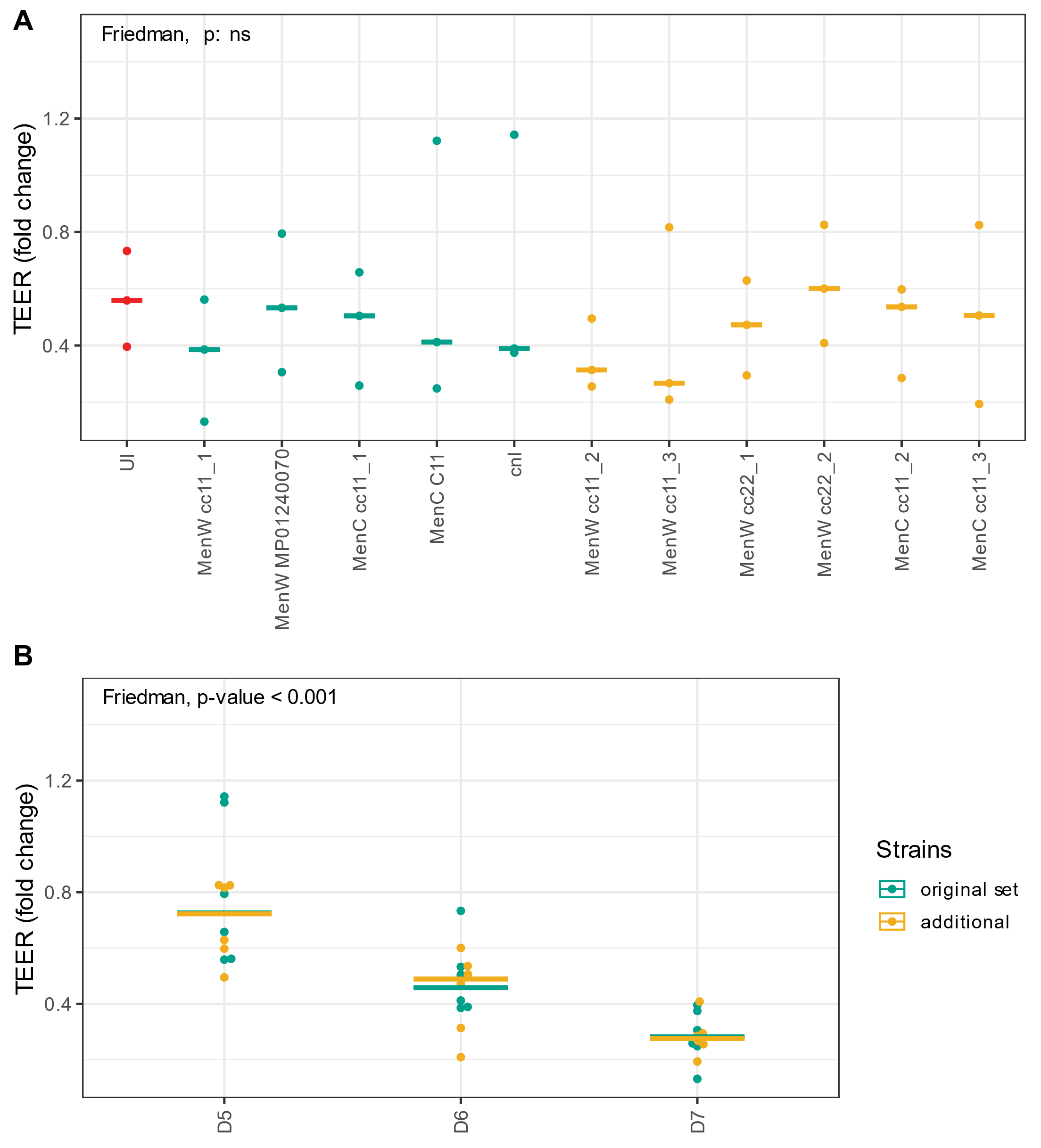


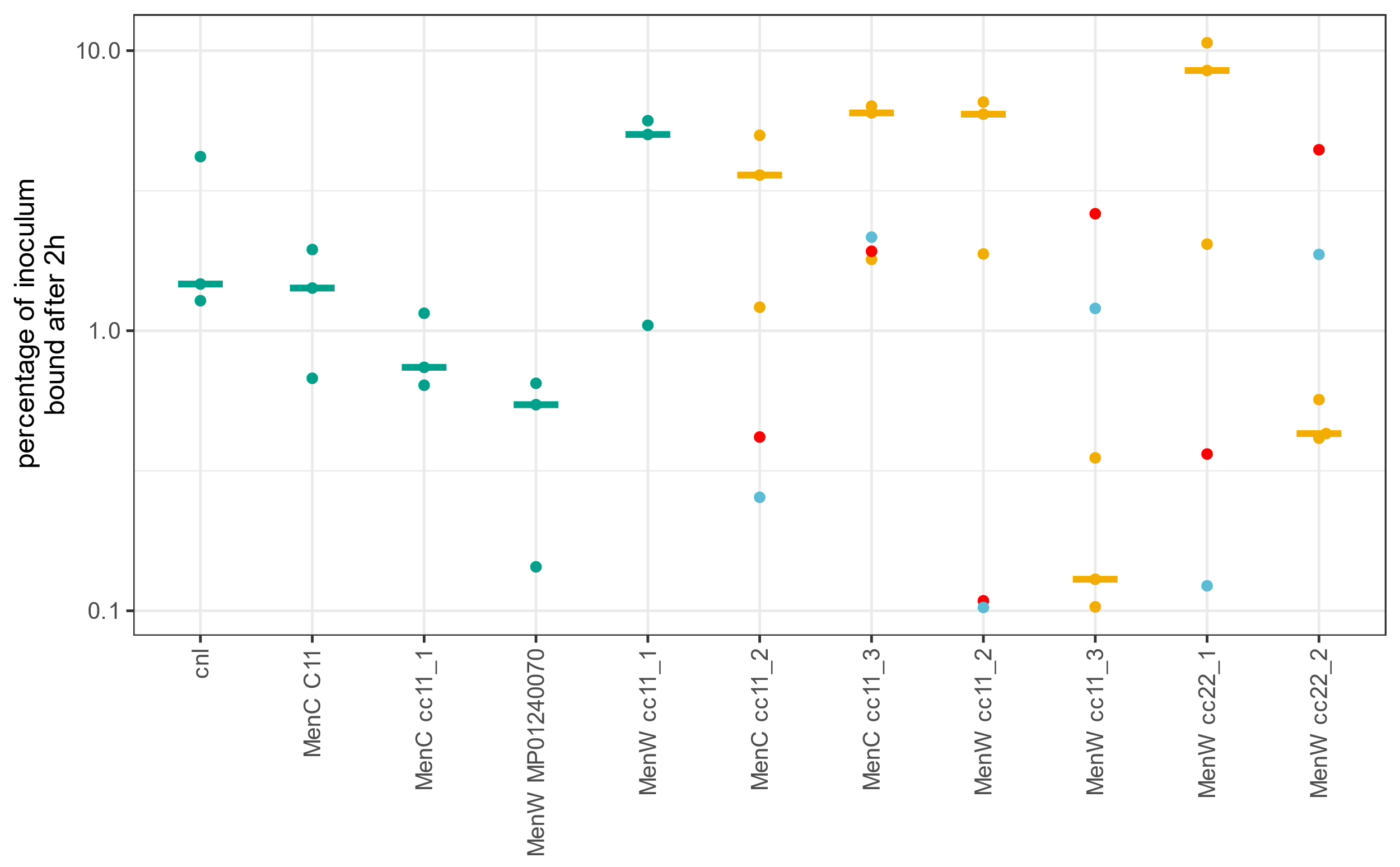
**Supplemental figure 2:** Binding of additional strains (yellow) at 2h post infection. High/low level binding on cell lines RPMI (red) and Calu-3 (light blue) did not translate into similar binding affinity on primary epithelium. Mean percentage bound for each donor with median is shown.

**Supplemental figure 3:** Donor specific cytokine inductions. For each condition, cytokine concentration at 6, 24 and 48h post infection are shown. Lines represent the mean geomean concentration across all donors with 95% confidence interval. Statistically significant differences were observed 24h post infection. Performed statistical analysis: per donor, the geomean concentration of individual strains was calculated. These were then compared using a permutation Friedman test stratified by strain.


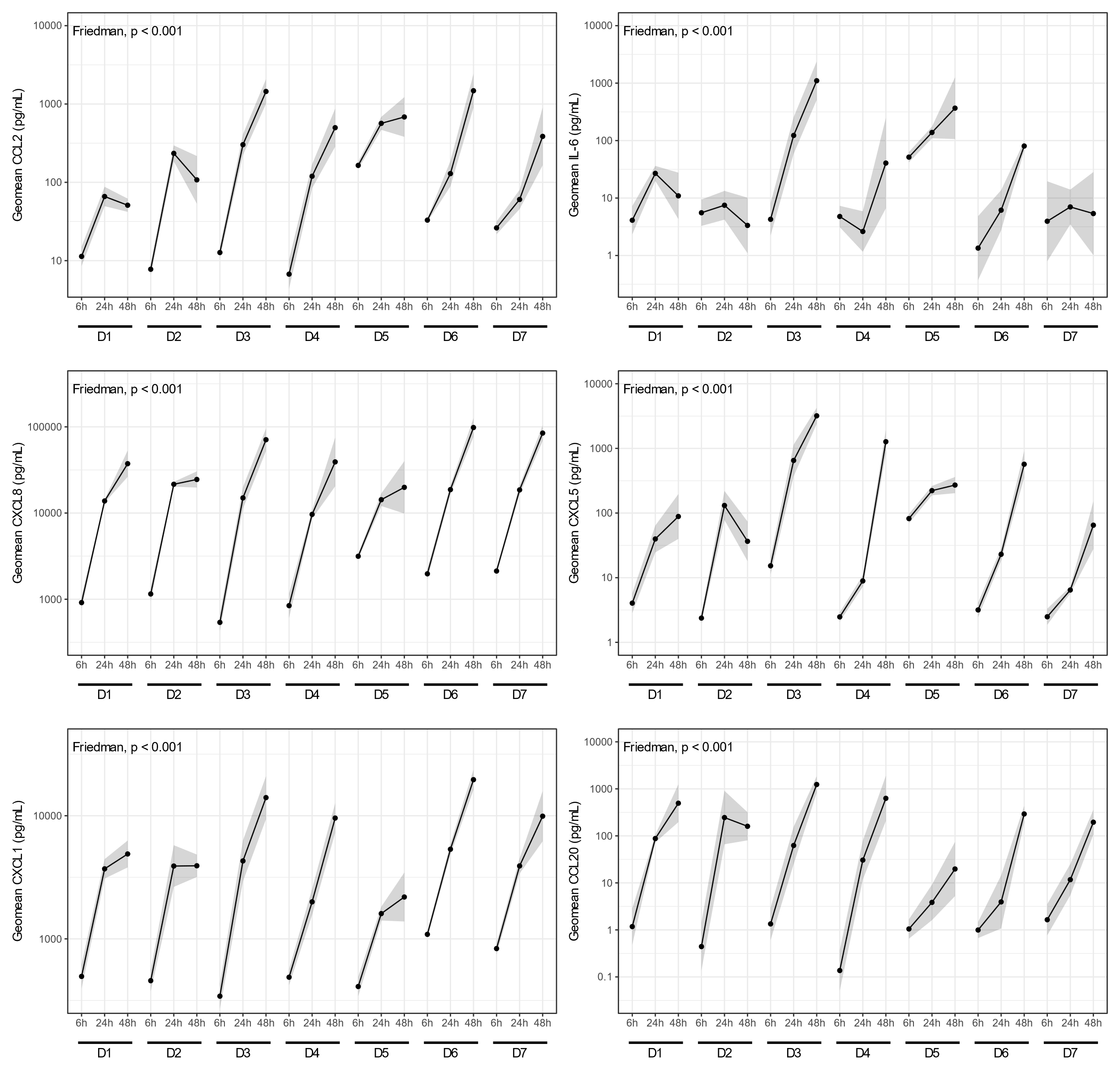

Supplement: Supplemental figures — Fig. S1 to S3. [file spectrum.00141-25-s0001.docx]
